# Effect of Iron Isomorphic Substitution in Mg:Al and Zn:Al-Layered Double-Hydroxide Structures by Means of First Principle Calculations

**DOI:** 10.1021/acsearthspacechem.2c00205

**Published:** 2022-10-04

**Authors:** Carlos Pimentel, Alfonso Hernández-Laguna, C. Ignacio Sainz-Díaz

**Affiliations:** Instituto Andaluz de Ciencias de la Tierra, Consejo Superior de Investigaciones Científicas-Universidad de Granada, Av. de las Palmeras, 4, 18100Armilla, Granada, Spain

**Keywords:** DFT, LDH, cation order, quantum mechanics, crystal structure

## Abstract

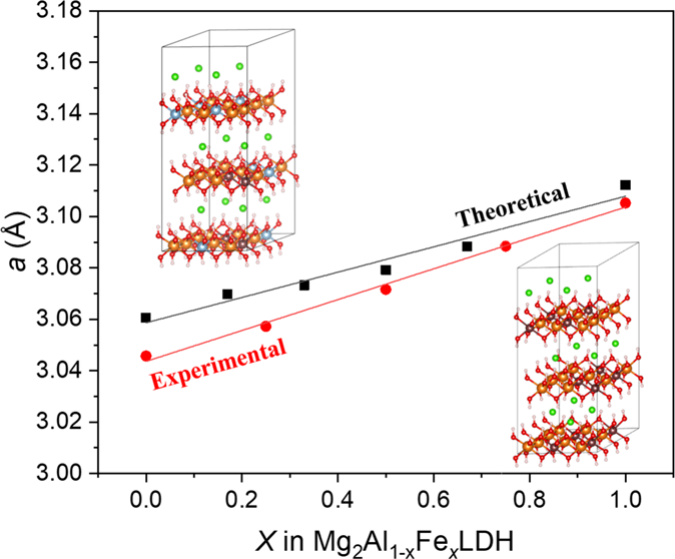

Layered double hydroxides (LDHs) are important components in terrestrial and extra-terrestrial environments. The presence of iron in these minerals provides them a wide potential application in environmental and materials sciences. In this work, the role of Fe in the crystallographic properties of LDHs M^2+^:M^3+^ 2:1 with Mg:(Fe,Al), Mg:Fe, Zn:(Fe,Al), and Zn:Fe is investigated by means of quantum mechanical calculations based on the density functional theory (DFT). Several relative proportions of Fe are studied. The cation ordering of these LDHs has been explored, finding useful insights for experimental synthetic paths of these minerals. The *a* and *b* cell parameters increase with the iron concentration. Some diffraction lines at high angle decrease in angle and increase in intensity with the increasing iron concentration. All of them agree with the experimental results. The iron substitutions tend to aggregate.

## 1 Introduction

Layered double hydroxides (LDHs) are metallic hydroxides with the crystal structure of brucite (Mg hydroxide). These hydroxide layers can have isomorphous cation substitutions in the octahedral sheet that in some cases can provoke charge defects, which are compensated with anions intercalated in the interlayer space. This fact gives the main properties of these minerals, which gives their high absorption capacity, variable water content, and anion exchanges. These capabilities and their small particle size make these minerals inorganic membranes with catalytic properties. Moreover, LDHs are present in seabed hydrothermal vents. In this scenario, LDHs provide confined spaces where the adsorption and their catalytic properties can become a possible scenario for the first prebiotic chemical reactions for the origin of life^1−3^ in early Earth.^4^

Furthermore, Fe hydroxides can also form LDHs with Mg, producing minerals such as pyroaurite or iowaite.^5^ These iron minerals can be present in the serpentinization processes of early Earth. The Mg–Fe layers of LDHs are more reactive than no-Fe-bearing LDHs due to the increasing basicity of the hydroxyl groups. In addition, the presence of iron in LDHs also provides redox reactions due to the Fe^3+^–Fe^2+^ binary system.

These Fe-bearing LDHs have also great medical and scientific interest. The iron content in LDHs increases the biocompatibility of these solids, helping in tissue regeneration by modulation of collagen production.^6^ On the other hand, some Fe-bearing LDH minerals have the Fe^2+^–Fe^3+^ pair in the structure, which are known as green rust, being the main mineral the so-called fougerite.^3,5,7^ This mineral has been particularly related to the origin of life; therefore, many efforts have been reported for understanding its structure and physicochemical properties.^5,7−9^

In addition, the cation ordering in LDHs is becoming an interesting issue, although different researchers have obtained contradictory results. Thus, some studies show that the cations in LDHs are distributed orderly,^10,11^ whereas others show that these cations are randomly distributed.^12−14^ In all cases, it was found that M^3+^ cations avoid close contact.^13,15^ Rozov et al.^16^ found crystallographic changes with the presence and content of Fe in MgFe-LDH without considering the Fe distribution along the crystal lattice, which has been subsequently demonstrated by Figueiredo et al.^17^ The Fe^3+^ cation distribution in the octahedral sheet of minerals can affect some properties, such as the nuclear magnetic resonance (NMR) and infrared spectroscopy properties in clay minerals.^18−20^

Taking into account the importance of Fe-bearing LDHs and cation ordering in LDHs, one of the aims of this work is to understand, at an atomic scale, the Fe cation distribution in LDHs and the variation of crystallographic properties of Mg:AlFe and Zn:AlFe LDHs with different relative proportions of Fe by means of first principle calculations.

## 2 Models

The LDH models used in this work were taken from our previous optimized theoretical models.^15^ In that paper, the structure of hydrotalcite,^21^ Mg_6_Al_2_(CO_3_)(OH)_16_·4H_2_O, was modified to design an LDH structure with a Mg^2+^:Al^3+^ ratio of 2:1, instead of 3:1, chlorine anions in the interlayer space instead of CO_3_^2–^ and removing the water molecules. Moreover, the Zn-LDH structure was also built by replacing Mg^2+^ cations with Zn^2+^ cations.

From the energetically most favorable optimized structure, a LDH 4 × 3 × 1 supercell was constructed for both Mg:Al-LDH and Zn:Al-LDH.^15^ The effect of Fe^3+^ cations in the LDH structure was studied by substituting different amounts of aluminum cations in the supercell Mg_24_(Al,Fe)_12_. These substitutions were always done in pairs up to a maximum of 12Fe^3+^ cations per supercell, which indicates the maximum amount of substitutions of trivalent cations in the supercell with a general formula Mg_24_Al_12–*x*_Fe*_x_*LDH, named in short as Mg:Al_12–*x*_Fe*_x_*. The same was done for Zn:Al-LDH with Zn_24_Al_12–*x*_Fe*_x_*LDH models, named Zn:Al_12–*x*_Fe*_x_*. All our models are dry, without any water molecules in the structure, to optimize the computational facilities and consider that this aspect does not alter the relative energy calculations between structures.

**Figure 1:**
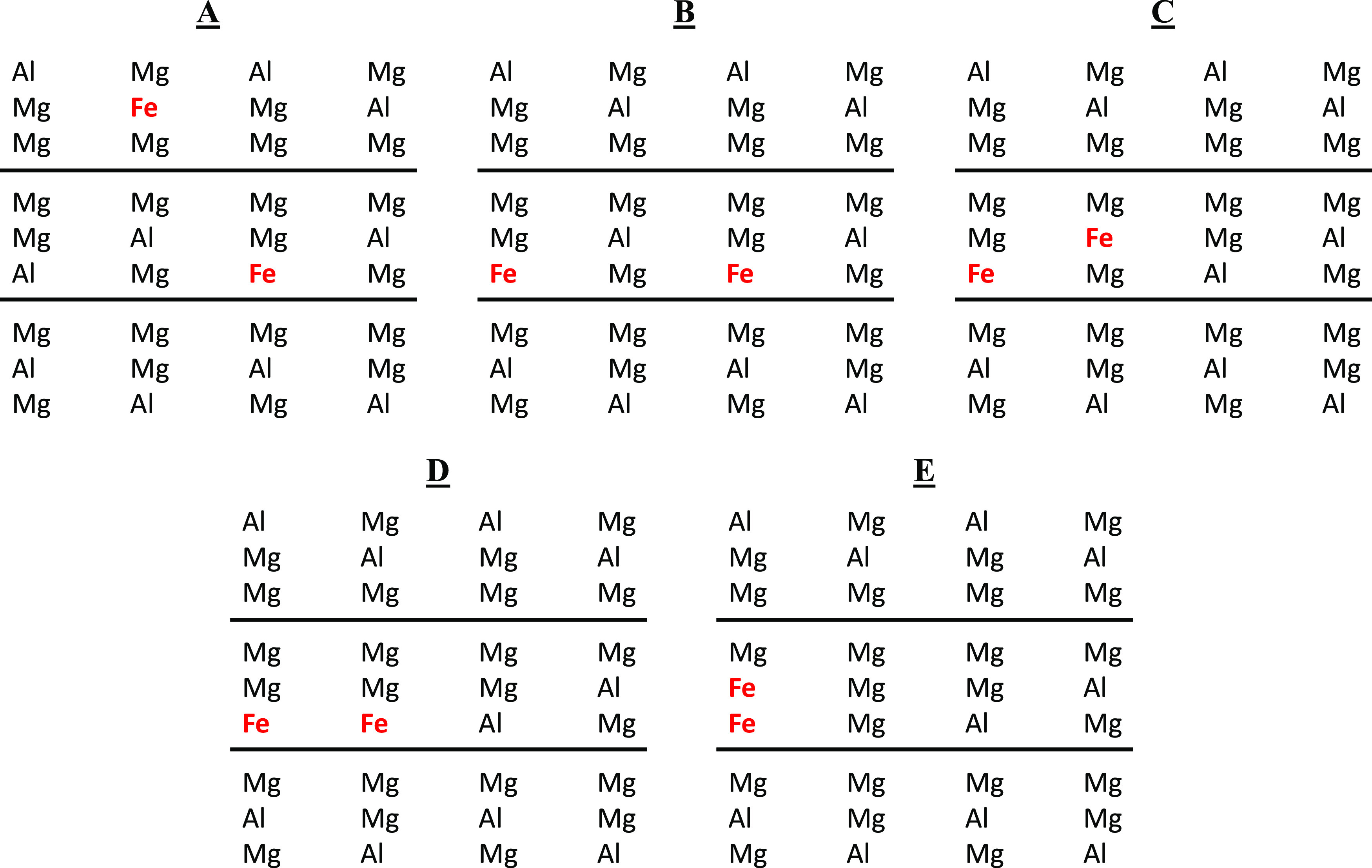
Scheme of the different cation distributions with two Fe^3+^ cations in the 4 × 3 × 1 supercell of LDH. (A) Separated in different layers (Mg:Al_10_Fe_2_DL), (B) placed in the same layer but in a separated configuration (Mg:Al_10_Fe_2_SL), (C) placed together along the [320] direction (Mg:Al_10_Fe_2_SLTa), (D) together along [100] (Mg:Al_10_Fe_2_SLTb), and (E) together along [010] (Mg:Al_10_Fe_2_SLTc). The lines indicate the separation between layers, which is composed of the OH groups bonded to the cations and chlorine anions placed in the interlayer space (these atoms are not shown to simplify the schemes).

Moreover, different Fe^3+^ cation arrangements were studied for Mg-LDH structures with 2Fe^3+^ cations, Mg_24_(Al_10_Fe_2_): (i) placed in different layers (Mg:Al_10_Fe_2_DL) (Figure 1A, DL means Fe^3+^ cations are distributed in different layers); (ii) both Fe^3+^ cations are placed separated in the same layer (Mg:Al_10_Fe_2_SL) (Figure 1B, SL means Fe^3+^ cations are distributed in the same layer); and (iii) both Fe^3+^ cations placed together in the same layer (Mg:Al_10_Fe_2_SLT) (Figure 1C, SLT means Fe^3+^ cations are distributed together in the same layer, forming a pair of cations). In addition, the disposition of the Fe^3+^ cations together can be placed following different orientations: (i) along [320] (Mg:Al_10_Fe_2_SLTa) (Figure 1C); (ii) [100] (Mg:Al_10_Fe_2_SLTb) (Figure 1D); and (iii) [010] (Mg:Al_10_Fe_2_SLTc) (Figure 1E).

## 3 Methods

The crystal structure and energies of the different LDH models were calculated using density functional theory (DFT). All these electronic calculations were carried out with the CASTEP code, implemented in the Materials Studio package,^22^ with the generalized gradient approximation (GGA) functional with the exchange and correlation functional of Perdew–Burke–Ernzerhof (PBE).^23^ All optimization calculations were performed using On-The-Fly-Generated (OTFG) ultrasoft pseudopotentials including the Koelling–Harmon relativistic treatment,^24^ Grimme dispersion correction,^25^ and an energy cutoff of 571.4 eV. The spin polarization was applied in two different ways, optimizing the spin state, or maintaining the minimal spin state. The Hubbard U correction^26,27^ was also taken into account with *U* = 2.5 eV in the d orbitals of Fe of the Mg_24_Al_12–*x*_Fe*_x_*LDH and Zn_24_Al_12–*x*_Fe*_x_*LDH crystals (*x* ≤ 12). The results, with and without these corrections, were compared in fully optimized crystal structures (i.e., atomic positions and cell parameters). The convergence tolerance parameters were set to 5 × 10^–6^ eV/atom for energy, 0.0001 Å for maximum displacement, 0.02 GPa for maximum stress, and 0.01 eV/Å for the maximum force. The convergence gradient for the self-consistent field (SCF) was 1 × 10^–7^ eV/atom in the density matrix. All of the crystal models shown in this work have been plotted using the Vesta software.^28^

Powder X-ray diffraction (XRD) patterns of the calculated structures were simulated with the REFLEX code included in Materials Studio.^22^ The simulation was carried out considering a copper X-ray source. Moreover, the samples were considered to have a crystallite size of 35 nm, which produces diffractograms more similar to those obtained experimentally in previous works.

## 4 Results and Discussion

As stated above in the model section, the Mg:Al and Zn:Al-LDH crystal structures used in this work were previously fully optimized by Pimentel et al.^15^ using the same theoretical procedure as described above. However, other parameters have to be taken into account in systems with Fe^3+^, i.e., spin polarization and Hubbard correction. Therefore, the best method of calculation had to be chosen prior to the in-depth study of the LDH structures with Fe^3+^. Then, we used the Mg-LDH with 12Fe^3+^ cations per supercell (Mg_24_Fe_12_) as a model structure (Mg:Fe_12_) (Table 1). Other approaches not shown in the table were used to optimize the LDH structure, such as no spin polarization and using free spin polarization without the Hubbard correction. However, after several trials, no convergence was obtained. Therefore, the best approach for calculating the LDH with Fe^3+^ is to use Hubbard correction with the optimized spin state (SP+U) and with the minimal net spin state equal to zero (SP0+U), whose optimized cell parameters are in good agreement with the previously reported experimental results^17^ (Table 1). The SP+U method yields the closest cell parameters to the experimental values. In addition, the crystal structure optimized with SP+U has 0.078 eV/uc (i.e., eV per unit cell) lower energy than SP0+U, indicating that the high spin state is slightly more stable than the paramagnetic low spin state. Although both methods have been used in this work, the main method used is SP+U, with which a larger amount of possible LDH structures with Fe^3+^ cations have been explored (including all of the Mg_24_Al_12–*x*_Fe*_x_*LDH and Zn_24_Al_12–*x*_Fe*_x_*LDH structures, Table 1). These results contrast with those previously obtained for Mg_24_Al_12–*x*_Fe*_x_*LDH bearing carbonate anions, in which the use of Hubbard correction was found to yield a slight overestimation of the lattice parameters.^29^

**Table 1:** Unit Cell Parameters of Mg_24_Fe_12_ Crystal Structures Calculated with the Minimal Net Spin State Equal to 0 (SP0), with Optimized Spin State and Hubbard Correction (SP+U), with the Minimal Net Spin State Equal to 0 and Hubbard Correction (SP0+U), and the Experimental Values^a^^17^

parameters	SP0	SP+U	SP0+U	exp
*a*	3.05	3.11	3.09	3.11
*b*	3.04	3.10	3.09	3.11
*c*	22.29	22.32	22.32	23.6
α	89.4	89.5	89.3	90.0
β	90.2	90.3	90.2	90.0
γ	120.1	119.9	119.9	120.0

a Distances are given in Å and angles in degrees.

The largest difference between the theoretical and experimental results is found in the *c* parameter, which could be due to the different amounts of water in the structure (our theoretical structure being anhydrous). This difference has also been observed in previous works.^30−32^

**Figure 2:**
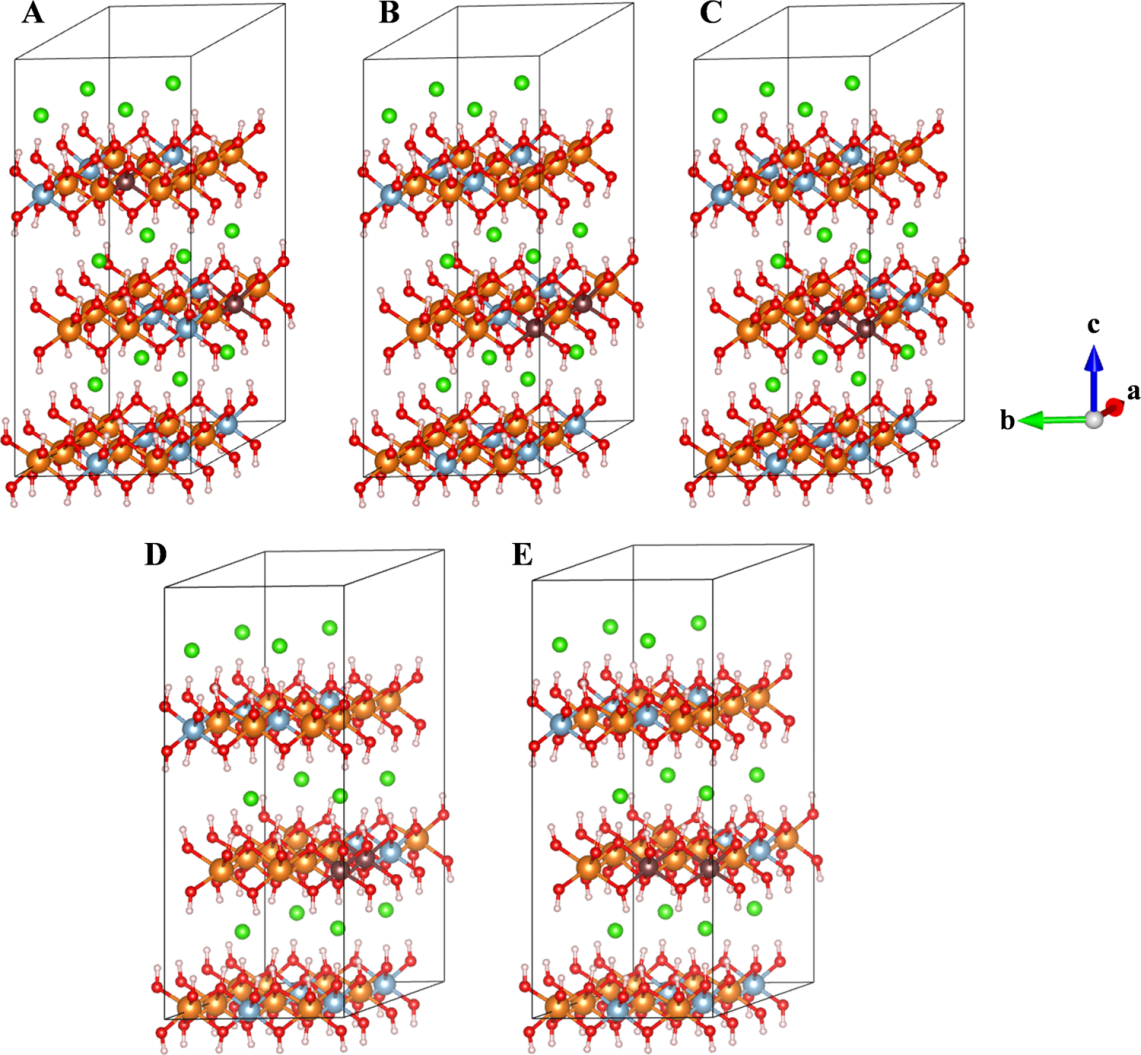
Optimized Mg:Al_10_Fe_2_-LDH crystal structures with different arrangements. (A) Fe^3+^ cations placed in different layers (Mg:Al_10_Fe_2_DL), (B) Fe^3+^ cations separated in the same layer (Mg:Al_10_Fe_2_SL), (C) Fe^3+^ cations placed together in the same layer along the [320] (Mg:Al_10_Fe_2_SLTa), (D) [100] (Mg:Al_10_Fe_2_SLTb), and (E) [010] (Mg:Al_10_Fe_2_SLTc) directions. Mg, Al, Fe, O, H, and Cl atoms are depicted in orange, light blue, brown, red, white, and green, respectively. This color representation is extended to the rest of this work.

Once the best calculation method has been determined, the Mg:Al_10_Fe_2_LDH structures with different arrangements of two Fe^3+^ were studied (Figure 1): placed in different layers (Mg:Al_10_Fe_2_DL), placed separated in the same layer (Mg:Al_10_Fe_2_SL), or placed together (Mg:Al_10_Fe_2_SLTa). After a full optimization of the geometry (i.e., lattice parameters and atomic positions), the geometry and the energy between the different structures were compared (Figure 2 and Table 2). No significant energy difference was observed between these structures. Considering the low energy differences per unit cell between these three models, it can be assumed that all of them could be equally found in experimental LDHs, although the distribution of iron cations in the LDH unit cell has not been studied experimentally so far.

**Table 2:** Unit Cell Parameters and Relative Energies of the Mg:Al_10_Fe_2_-LDH Crystal Structures with Different Fe^3+^ Arrangements^a^

parameters	Mg:Al_10_Fe_2_DL	Mg:Al_10_Fe_2_SL	Mg:Al_10_Fe_2_SLTa [320]	Mg:Al_10_Fe_2_SLTb [100]	Mg:Al_10_Fe_2_SLTc [010]
*a*	3.069	3.069	3.070	3.070	3.080
*b*	3.058	3.058	3.059	3.060	3.072
*c*	22.262	22.262	22.261	22.274	22.546
α	89.3	89.5	89.4	89.7	89.4
β	90.1	90.1	90.1	90.0	90.2
γ	120.3	120.3	120.3	120.4	120.4
energy	–0.003	–0.003	0.000	0.025	0.050

a Distances are given in Å, angles in °, and relative energy per unit cell in eV.

The next step to study the Mg-LDH structures with two Fe^3+^ cations was to determine the effect of the orientation of the Fe^3+^ cation pairs in the Mg:Al_10_Fe_2_SLT arrangements along [320], i.e., diagonal (Mg:Al_10_Fe_2_SLTa), [100] (Mg:Al_10_Fe_2_SLTb), and [010] (Mg:Al_10_Fe_2_SLTc) directions (Figure 1c–e, respectively), optimizing without any constraint. Only the structure with the iron pair aligned along the [010] direction shows a slight difference in the lattice parameters (Table 2). The difference in energies between the three arrangements is higher than those between DL and SL. Although the energy differences between these arrangements are not significant enough, the lowest-energy structure (Mg:Al_10_Fe_2_SLTa) is chosen as the default cation arrangement to build and calculate the structures with a higher amount of Fe^3+^ cations.

**Figure 3:**
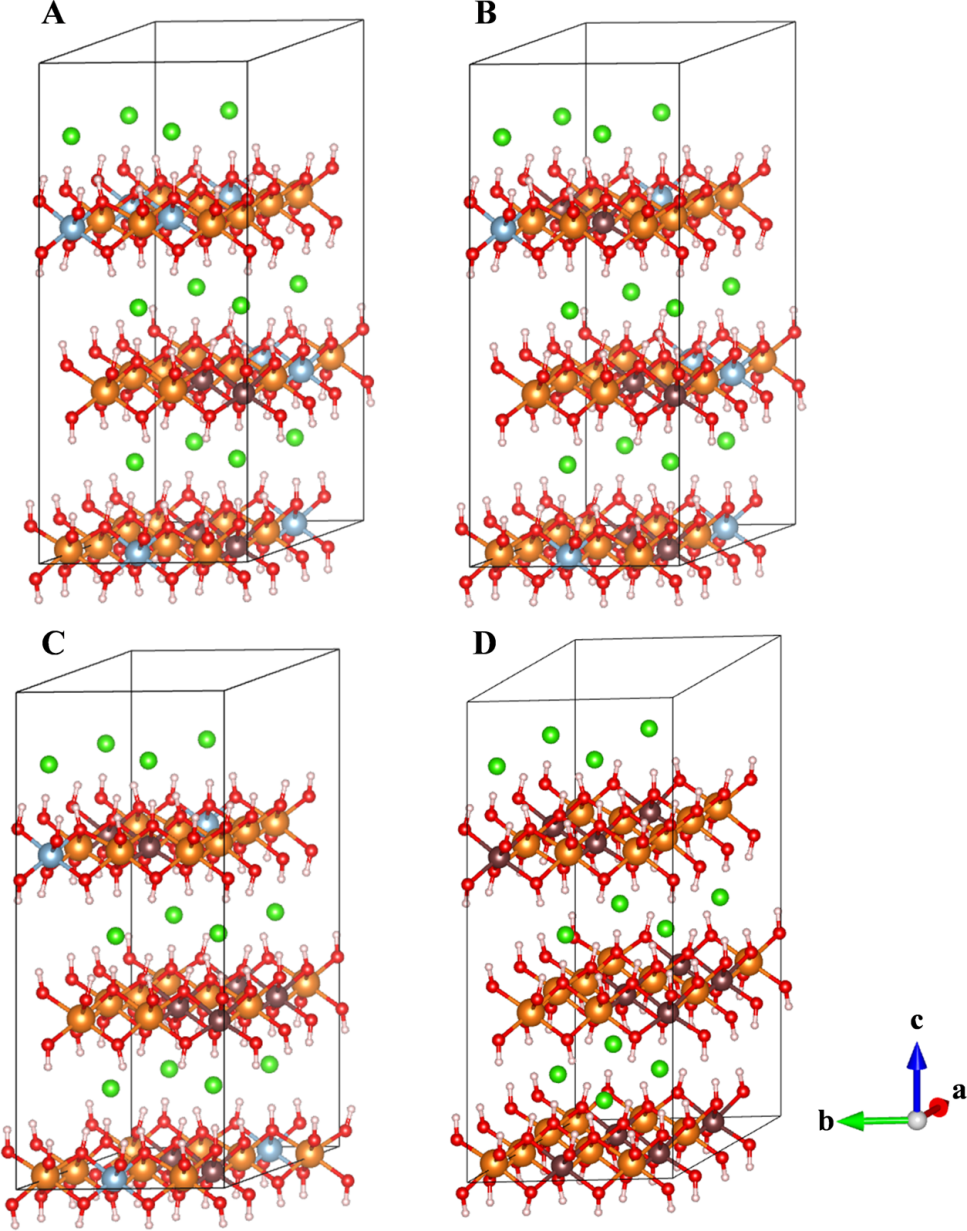
Structures of Mg-LDH bearing 4 (A), 6 (B), 8 (C), and 12 (D) Fe^3+^ cations per 4 × 3 × 1 supercell.

In the Mg:Al_10_Fe_2_SLTa model, two Al^3+^ cations were replaced with additional two Fe^3+^ cations, being together in the next layer with respect to the previous Fe^3+^ cation pair (Mg:Al_8_Fe_4_DL) or being all Fe^3+^ cations together in the same layer (Mg:Al_8_Fe_4_SL). In the same way, two additional Fe^3+^ cations were substituted, placing them together but in another layer (Mg:Al_6_Fe_6_), where two Fe^3+^ cations formed pairs in each layer in the 2–2–2 sequence along the *c*-axis. From this structure, two additional Al^3+^ cations of the central layer were substituted by two Fe^3+^ cations (Mg:Al_4_Fe_8_), with the 2–4–2 sequence of the Fe^3+^ cations in the layers of the supercell along the *c*-axis. Finally, all Al^3+^ cations were substituted by Fe^3+^ cations in the structure described above (Mg:Fe_12_). All these LDH crystal structures were fully optimized, i.e., atomic positions and lattice parameters (Figure 3 and Table 3). The model with 4Fe^3+^ cations together in the same layer (Mg:Al_8_Fe_4_SL) is 0.061 eV/uc less stable than that with the Fe^3+^ cation pairs in different layers (Mg:Al_8_Fe_4_DL).

**Table 3:** Unit Cell Parameters and Energies of the Mg-LDH Crystal Structures with Different Amounts of Fe^3+^ Cations (2, 4, 6, 8, and 12 per 4 × 3 × 1 Supercell)^a^

parameters	Mg:Al_10_Fe_2_SLTa	Mg:Al_8_Fe_4_DL	Mg:Al_8_Fe_4_SL	Mg:Al_6_Fe_6_	Mg:Al_4_Fe_8_	Mg:Fe_12_
*a*	3.070	3.073	3.068	3.079	3.088	3.112
*b*	3.059	3.068	3.058	3.074	3.082	3.105
*c*	22.261	22.298	22.278	22.298	22.306	22.316
α	89.4	89.6	89.4	89.4	89.5	89.5
β	90.1	90.1	90.2	90.2	90.1	90.3
γ	120.3	120.3	120.3	120.1	120.1	119.9
energy	–6789.6289	–6913.9565	–6913.9054	–7038.2345	–7162.5870	–7411.2842

a Distances are given in Å, angles in degrees, and energies (*E*) in eV per unit cell.

Hypothetical isodesmic reactions can be designed to explore some significant substitutions. The isodesmic reactions have the advantage of having the same structure, same bonds, and same number and type of atoms either in reactants or products. All errors due to the electron basis set, correlation exchange functionals, pseudopotentials, and different approximations for calculating the electronic structure and optimal atomic distances and the cell parameters would be canceled. This approach has been applied previously in octahedral cation substitutions in phyllosilicates^33^ and organic reactions.^34^

Three reactions have been designed by simplifying the names of components (Fe_12_ instead of Mg:Fe_12_)
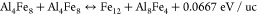
1
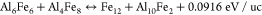
2
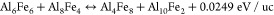
3All of them are exothermic reactions, giving special stability to products, where there are compounds with the largest amount of Fe aggregated. Nonetheless, these energy differences are higher than those from the Fe ordering of the samples with 2Fe cations per supercell (Mg:Al_10_Fe_2_SL and Mg:Al_10_Fe_2_DL) (Table 2). From these hypothetical reactions, we find that the more stable combinations are those in which one compound has the maximum aggregated Fe yield. We can conclude that the Fe cations tend to be aggregated. Nevertheless, these energy differences are not significant, meaning that different combinations can be formed during the synthesis process while maintaining the same global iron content, although Fe cations tend to aggregate. This fact would seem the opposite of what was highlighted between Mg:Al_8_Fe_4_SL and Mg:Al_8_Fe_4_DL arrangements; however, in the former, all Fe cations are distributed in the same single layer, leaving two layers up and down without any substitution in our model. As a consequence, certain distortion is created between the layers, introducing an additional destabilization in the structure.

**Figure 4:**
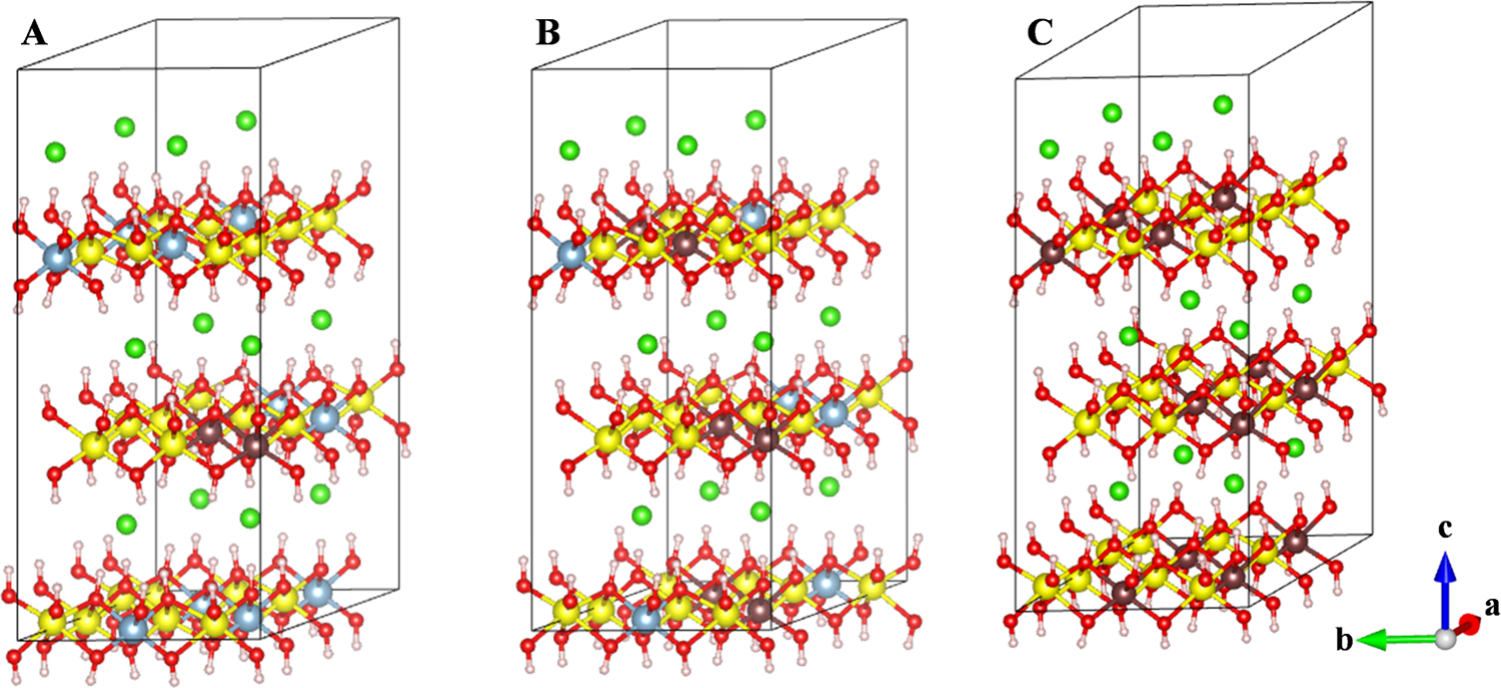
Optimized structures of Zn-LDH with 2 (A), 6 (B), and 12 (C) Fe^3+^ cations. Zn atoms are represented in yellow.

A similar tendency is found in the Zn-LDH structures bearing 2 (Zn:Al_10_Fe_2_), 6 (Zn:Al_6_Fe_6_), and 12 (Zn:Fe_12_) Fe^3+^ cations per supercell (Figure 4). In such structures, the energy difference between Zn:Fe_12_ and Zn:Al_6_Fe_6_ was only 0.0629 eV/uc lower than that between Mg:Fe_12_ and Mg:Al_6_Fe_6_. In the same way, the energy difference between Zn:Fe_12_ and Zn:Al_10_Fe_2_ is slightly higher, 0.0014 eV/uc, than that between Mg:Fe_12_ and Mg:Al_10_Fe_2_.

**Figure 5:**
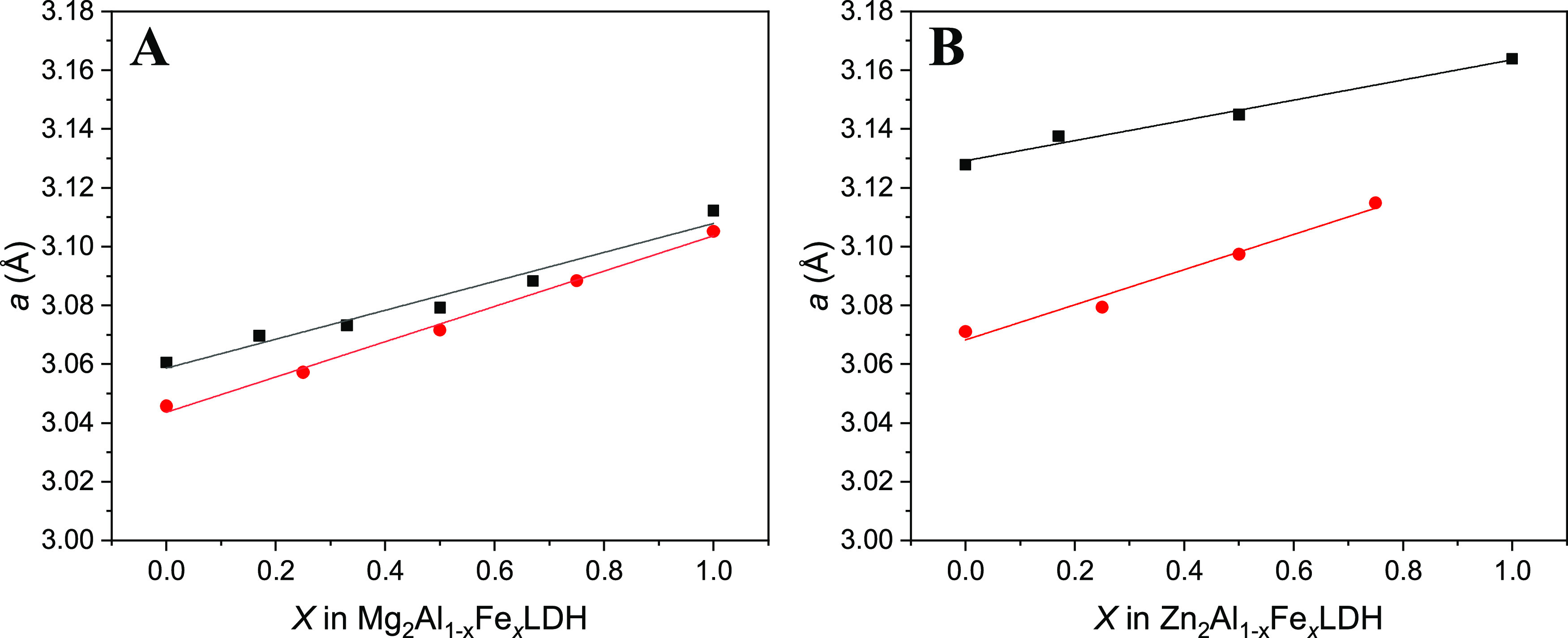
Variation in the unit cell *a* lattice parameter of Mg-LDH (A) and Zn-LDH (B) as a function of the relative proportion of Fe^3+^ cations from DFT calculations (black squares) and previously reported experimental results from Figueiredo et al.^17^ (red circles).

The size of the lattice parameters increases with the increasing amount of Fe^3+^ cations in the Zn-LDH structures (Tables 3 and 4), following linear relationships (Figure 5), similar to Mg-LDH. This behavior of the LDH structures has also been observed in Mg- and Zn-LDH crystals synthesized experimentally in the laboratory.^16,17^ By comparing both theoretical and experimental results (Figure 5), we find that the computational method used here not only provides crystal structures with cell parameters in good agreement with those previously reported but also reproduces a slight change in the *a*/*b* cell axes measured in synthesis experiments with different iron contents. This behavior was also observed experimentally in LDH hydrotalcite–pyroaurite systems Mg(AlFe), showing the same slope (0.05) of these linear relationships.^16^ This can be explained by the longer Fe–O bond distance, d(Fe–O) = 2.03 ± 0.08 Å than the Al–O bond, d(Al–O) = 1.93 ± 0.04 Å, in both Mg_24_Al_12–*x*_Fe*_x_*LDH and Zn_24_Al_12–*x*_Fe*_x_*LDH systems. This can be due to the greater size of Fe^3+^ than that of Al^3+^ cations.

**Table 4:** Unit Cell Parameters and Energies of the Zn-LDH Crystal Structures with Different Amounts of Fe^3+^ Cations^a^

parameters	Zn:Al_10_Fe_2_	Zn:Al_6_Fe_6_	Zn:Fe_12_
*a*	3.138	3.145	3.164
*b*	3.120	3.134	3.163
*c*	22.178	22.175	22.184
α	89.5	89.2	89.3
β	90.0	90.1	90.0
γ	120.8	120.5	120.2
energy	–6977.5394	–7226.2093	–7599.1961

a Distances are given in Å, angles in degrees, and energies are in eV per unit cell.

**Figure 6:**
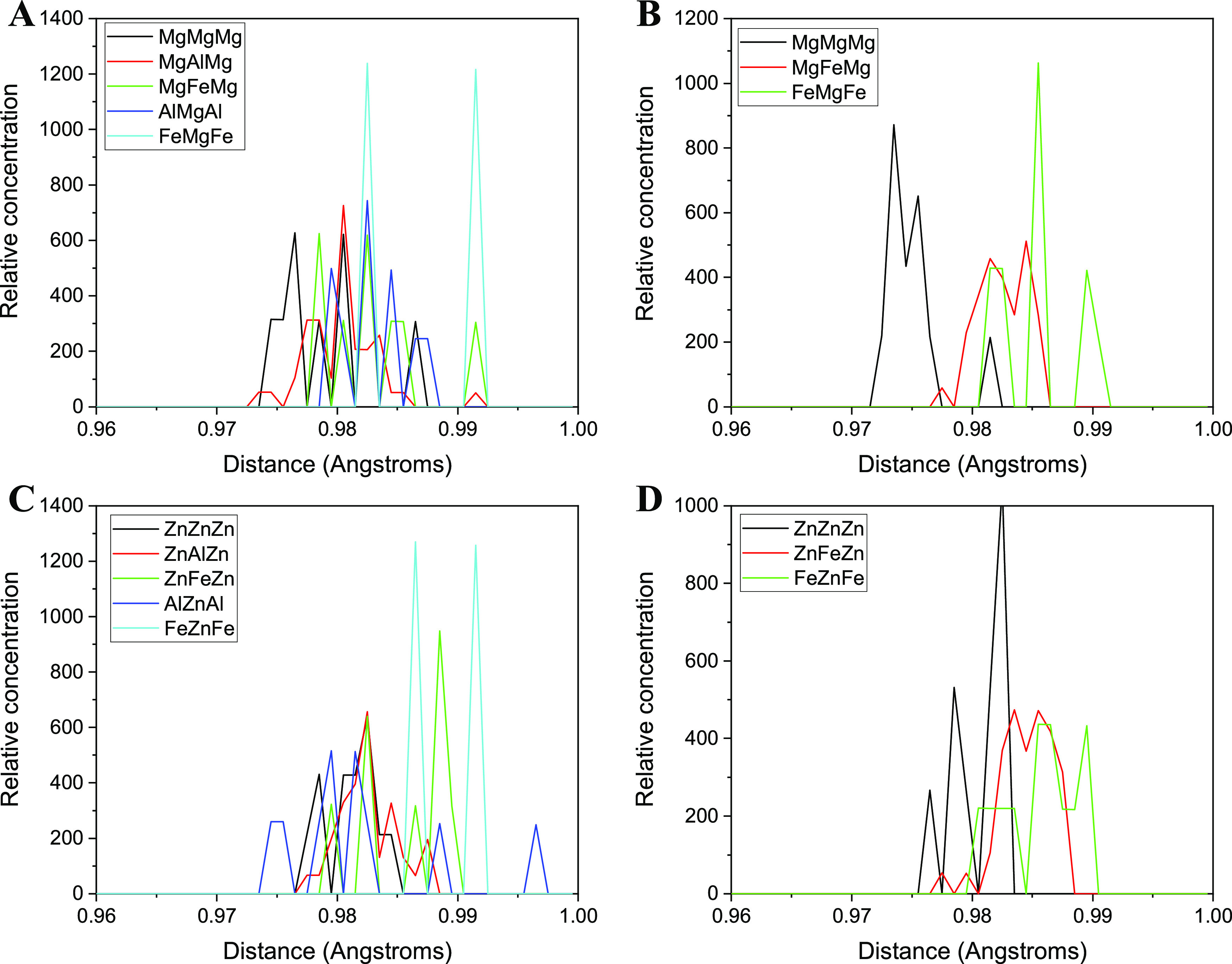
Radial distribution function of O···H bonds in Mg/Zn:Al_12–*x*_Fe*_x_*LDH structures: (A) Mg:Al_10_Fe_2_SLTa, (B) Mg:Fe_12_, (C) Zn:Al_10_Fe_2_SLTa, and (D) Zn:Fe_12_. The legend indicates the nature of the three cations to which the O atoms are bonded.

In addition to Fe–O and Al–O bonds, the O–H bonds were also measured, distinguishing the cations to which the O atoms are linked (Table 5 and Figure 6). In all LDH structures, the d(O–H) linked to FeXFe, X either being Mg or Zn, is the longest bond, whereas the shortest ones are those linked to three magnesium or zinc cations. In both LDH structures, the length of the O–H bonds can be considered to be similar when O atoms are linked to one or two aluminum cations or only to one iron cation. In general, the O–H bond lengths increase with the relative proportion of Fe^3+^. Exploring the radial distribution function of the neighbors O and H atoms, we can observe the short distances, which indicate the different values of the O–H bond lengths (Figure 6). Small differences can be observed due to the nature of the cation joined to them. In general, the ZnZnZn OH groups are longer than the MgMgMg ones. In the X:Fe_12_ (X = Mg or Zn) models, the d(O–H) bond length follows the sequence XXX < XFeX < FeXFe, although the differences are smaller in the models with Zn than those with Mg. However, in the X:Al_10_Fe_2_SLTa models, the differences are diffused and not so sharp, indicating that other factors are involved, such as nonbonding interactions with the Cl^–^ anions, and orientation of the OH bonds in the interlayer space. A similar phenomenon was observed previously in the octahedral sheet of phyllosilicates, which produces a shift to lower infrared frequencies in the vibration modes of these OH groups when the Fe^3+^ are joined to these OH groups.^35,36^

**Table 5:** O–H Bond Length in MgFe-LDH and ZnFeLDH Structures^a^

	Mg:Al_10_Fe_2_SLTa	Mg:Fe_12_	Zn:Al_10_Fe_2_SLTa	Zn:Fe_12_
XXX	0.979 ± 0.004	0.976 ± 0.003	0.981 ± 0.004	0.980 ± 0.002
XAlX	0.981 ± 0.005		0.983 ± 0.003	
XFeX	0.984 ± 0.005	0.982 ± 0.003	0.985 ± 0.004	0.984 ± 0.003
AlXAl	0.984 ± 0.003		0.982 ± 0.007	
FeXFe	0.987 ± 0.006	0.986 ± 0.004	0.987 ± 0.006	0.985 ± 0.003

a The first column indicates the nature of the three cations to which O is bound. X indicates Mg or Zn cations, depending on the structure considered. Distances are given in Å.

**Figure 7:**
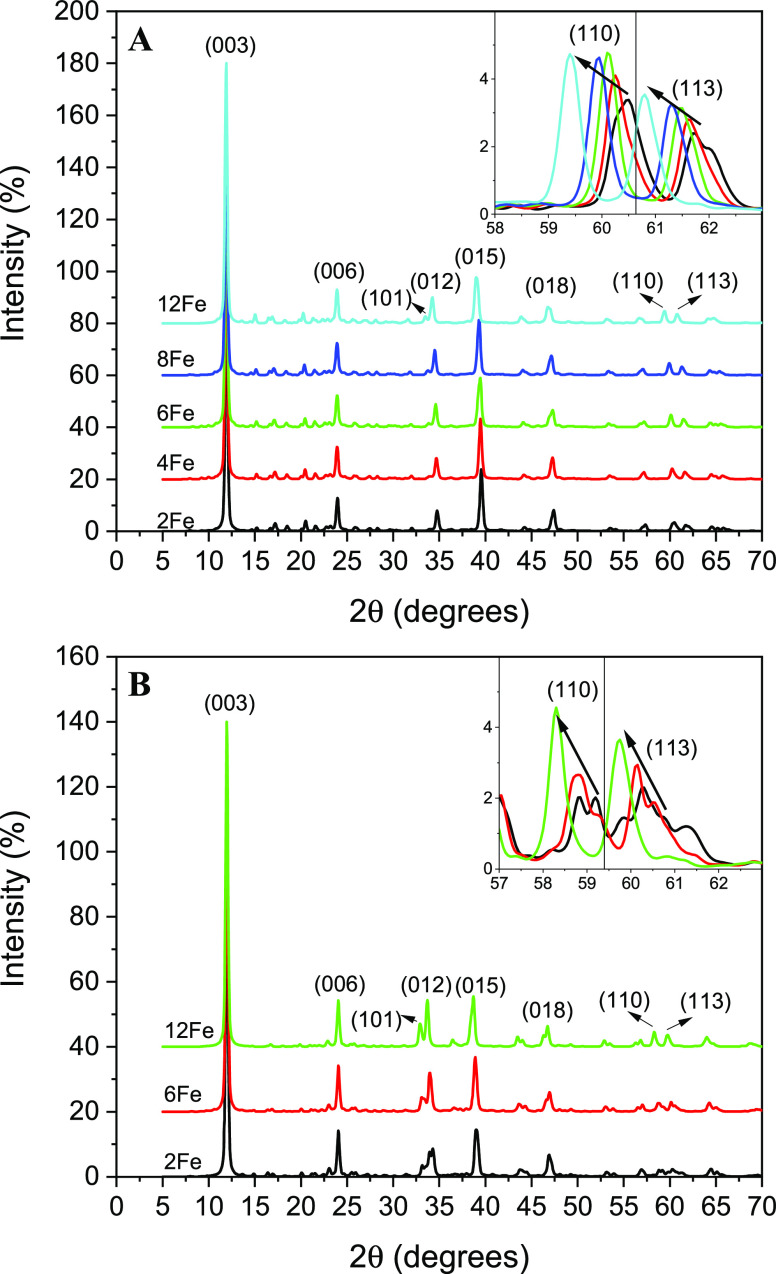
Theoretical diffractograms of (A) FeMgLDH and (B) FeZnLDH. LDH peaks have been identified. In the insets, closed views of the 57–63° region are shown. In the inset, it can be seen how the increment of the Fe cations in the LDH structure changes the position of the (110) and (113) peaks to lower 2θ degrees. Between each diffractogram, an offset of 20% has been set to allow better visualization. In each diffractogram, the (003) maximum corresponds to 100%. The labels 2Fe, 4Fe, 6Fe, 8Fe, and 12Fe correspond to the X:Al_10_Fe_2_SLTa, X:Al_8_Fe_4_, X:Al_6_Fe_6_, X:Al_4_Fe_8_SLTa, and X:Fe_12_ models, respectively.

For each calculated structure, the diffractograms were also simulated (Figure 7). The diffractograms are similar to those experimentally obtained in the previous works on the LDH structures.^16,17,32^ Taking into account that no symmetry was imposed in these calculations, some small peaks appear in the calculated diffractograms, in addition to the main LDH peaks that cannot be assigned to any LDH plane, which are spurious planes produced for considering a small number of unit cells. In our theoretical diffractograms (Figure 7), the positions of the (003) and (006) peaks are quite close for the different amounts of Fe cations considered; for example, in the case of the (003) peak, the positions are around 11.9° for both MgFe-LDH and ZnFeLDH structures. The main differences in the diffractograms are those related to the (110) and (113) peaks. In these cases, structures with higher iron content have lower 2θ angles (Figure 7). In MgFe-LDH structures, the (110) peak is placed at 60.5° for Mg-LDH (without any iron), and its position moves to lower spaces up to 59.4° for Mg:Fe_12_, while the position of the (113) peak changes from 62 to 60.8°. In the ZnFeLDH structures, the same differences have been observed, changing the positions of the peaks from 59.2 to 58.3° for (110) and from 61.3 to 59.7° for (113) by increasing the Fe content. Figueiredo et al.^17^ described the same behavior of the (110) and (113) peaks with the increasing amount of iron in the structure, i.e., their position moved to lower 2θ angles. This result also confirms the goodness of our calculations, since in addition to reproducing the structure, the same behavior has been observed with the increase of the iron content in the LDH structure, as described in previous works, i.e., the increasing the parameter *a* and *b* axes and the displacement toward lower angles of the peaks (110) and (113) in the diffractograms.

## 5 Conclusions

Our DFT calculations reproduce the crystal structures of Mg:Al_12–*x*_Fe*_x_*-LDH and Zn:Al_12–*x*_Fe*_x_*-LDH with different amounts of Fe^3+^ cation. The high spin states are the most probable in these systems. A certain preference for clustering of Fe^3+^ cations can be observed in these systems; however, this tendency is not clear probably due to the high content of Mg. Similar behavior was observed in phyllosilicates with a high proportion of Mg.^20^

These DFT calculations have reproduced the linear relationships of the cell parameters increasing with the relative proportion of Fe^3+^ cations observed experimentally in both systems, Mg-LDH and Zn-LDH. The in-depth study of the structural parameters of the calculated LDHs has allowed us to determine that the presence of iron increases the length of the bonds to which it is linked, affecting both M–O and O–H bonds.

Moreover, the displacement of the (110) and (113) peaks to lower 2θ angles with the increasing amount of iron in the LDH structure have been also observed, reproducing the experimental results previously reported.

This work opens the door for further theoretical studies on Fe-bearing LDHs, including fougerite, which could be of great interest for research studies related to the origin of life.
